# Evaluation of buccal damage associated with acute inhalation exposure to 2,4-dichlorophenoxyacetic acid (2,4-D) in mice

**DOI:** 10.1186/s12917-020-02461-w

**Published:** 2020-07-14

**Authors:** José Luiz Santos Parizi, Amanda Jodas Tolardo, Ana Carolina Gomes Lisboa, Bruna Barravieira, Fabíola de Azevedo Mello, Renata Calciolari Rossi, Gisele Alborghetti Nai

**Affiliations:** 1 Department of Pathology, Universidade do Oeste Paulista (UNOESTE), 19050-680 Presidente Prudente, SP Brazil; 2 Faculty of Dentistry, Universidade do Oeste Paulista (UNOESTE), SP 19050-680 Presidente Prudente, Brazil; 3 Graduate Program in Animal Science, Universidade do Oeste Paulista (UNOESTE), SP 19050-680 Presidente Prudente, Brazil; 4 Department of Pathology, Graduate Program on Environment and Regional Development, Universidade do Oeste Paulista (UNOESTE), SP 19050-680 Presidente Prudente, Brazil; 5 Department of Pathology, Graduate Program in Animal Science, Universidade do Oeste Paulista (UNOESTE), 19050-680 Presidente Prudente, SP Brazil; 6 Laboratório de Anatomia Patológica e Citopatologia, Universidade do Oeste Paulista (UNOESTE), Rua José Bongiovani, 700, SP 19050-680 Presidente Prudente, Brasil

**Keywords:** Leukoplakia, oral, Inflammation, Mast cells, Pesticide exposure, Environmental exposure

## Abstract

**Background:**

The herbicide dichlorophenoxyacetic acid (2,4-D) is one of the most widely used crop spraying products in the world. Some pesticides induce the degranulation of mast cells and increase allergic responses. This is the first study to evaluate the damage to the oral mucosa after an experimental simulation of environmental inhalation exposure to the 2,4-D herbicide. The aim of this study was evaluate the possible oral damage caused by acute inhalation exposure to the herbicide 2,4-D.

**Results:**

There was a difference between the exposure concentrations in relation to tissue congestion intensity (*p* = 0.002) and mast cell counts (*p* = 0.002), a difference in the evaluation of the interaction between the exposure concentrations and nebulization time in the dorsum epithelium thickness (*p* = 0.013), and a significant correlation between the epithelial thickness and the number of nucleoli organizing regions on the dorsum of the tongue (*p* = 0.048).

**Conclusions:**

Even after acute exposure, the herbicide 2,4-D had the potential to damage the oral epithelium, especially at higher doses.

## Background

Acid herbicides are an important class of pesticides, of which 2,4-dichlorophenoxyacetic acid (2,4-D) (C_8_H_6_Cl_2_O_3_) stands out because of its prevalent global use. This compound has been used to control a variety of weeds in cereal crops, sugarcane, and orchards, and it is also used for forest control [1, 2].

The herbicide 2,4-D belongs to the class of phenoxyacetic acids and has been used since the Vietnam War, when it was used by the United States Air Force as a defoliant agent along with 2,4,5-trichlorophenoxyacetic acid (2,4,5-T) and pentachlorophenol to form “Agent Orange”. This herbicide has replaced manual and mechanical weeding, resulting in an increased agricultural production [1]. In addition, 2,4-D is the herbicide that is most commonly used to kill weeds on lawns [3], and it is used not only in the rural environment but also in the squares and domiciliary gardens of the urban environment.

Some studies have shown chromatin damage to the oral cavity cells in the inhabitants of cities in Vietnam that were heavily bombarded with “Agent Orange” in the 1960s [4]. In addition, studies have demonstrated a higher prevalence of lesions of the buccal and labial mucosa and a greater chance of developing exfoliative cheilitis, hyperkeratosis of the tongue, lips and buccal mucosa, and leukoplakia in workers who manufacture herbicides in the chlorophenoxide class [5].

The herbicide 2,4-D is one of the most widely-used crop spraying products in the world [6]. The primary route of 2,4-D exposure, both occupational and para-occupational, is inhalation [6]. The mucosa of the oral cavity, which is close to the respiratory tract mucosa, is one of the first areas that is contaminated by agrochemicals due to the formation of mist during spraying. This is the first study to evaluate the buccal mucosa in an experimental simulation of environmental inhalation exposure (occupational and para-occupational) to the herbicide 2,4-D at different concentrations that are similar to those used in crops.

The aim of this study was to evaluate the effect of acute inhalation exposure to the herbicide dichlorophenoxyacetic acid (2,4-D) on the buccal mucosa of mice.

## Results

All data collected during the experiment were included in this study.

### Histopathological analysis

No hyperkeratosis, parakeratosis, individual cell necrosis or dysplastic or neoplastic lesions were observed in any of the groups studied.

**Fig. 1:**
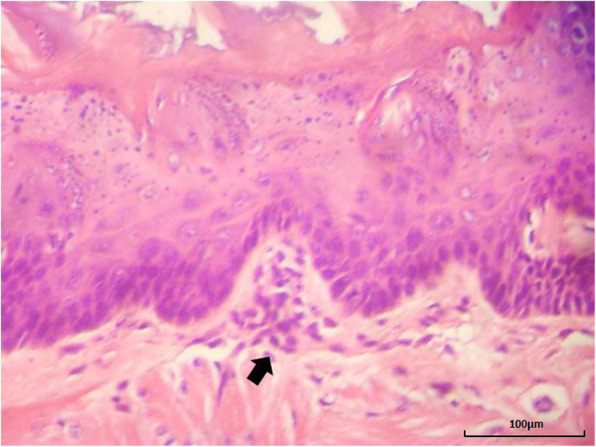
Photomicroscopy of the tongue with inflammatory foci in the submucosa (arrow). Hematoxylin-eosin, 400x magnification (100 µm bar for scale)

Two animals in the SG and one in the MCG presented with a moderate inflammatory process with the presence of mononuclear cells. The other animals presented with a mild inflammatory mononuclear infiltrate (*p* = 0.200) (Fig. 1).

**Fig. 2:**
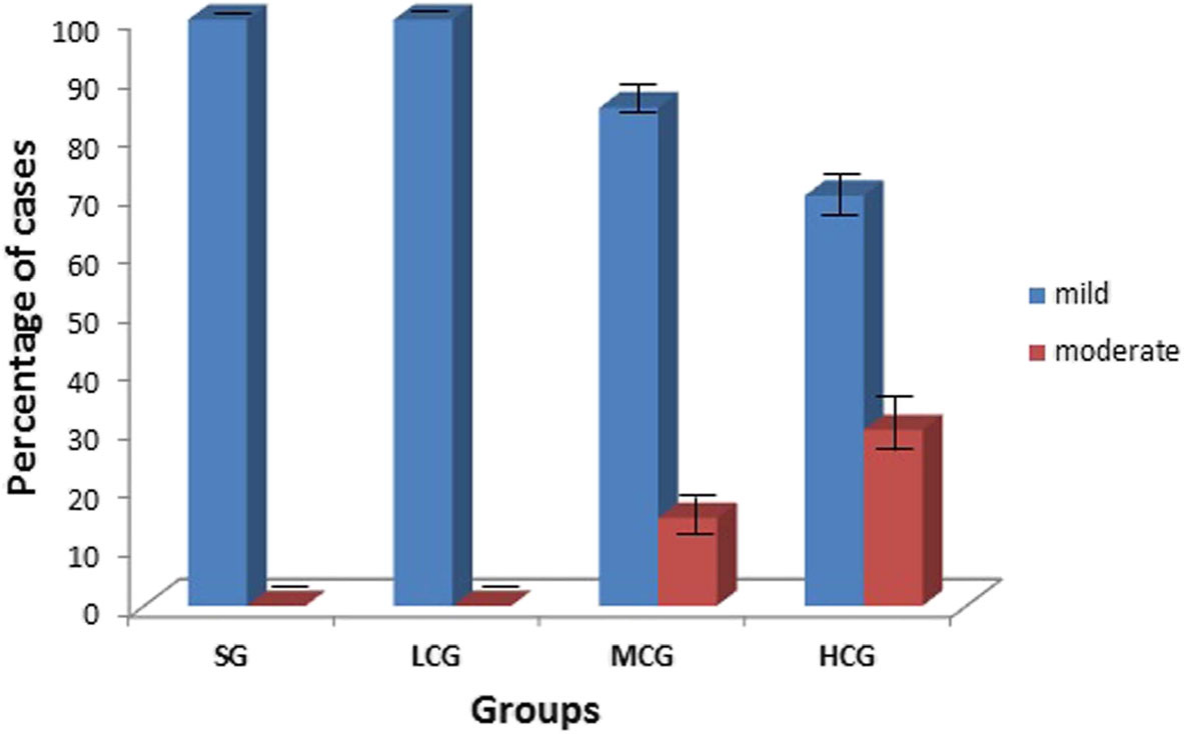
Intensity (percentage ± standard error) of congestion in the tongue of the animals per study group regardless the time of exposure. Groups: SG: saline group; LCG: low 2,4-D concentration group; MCG: middle 2,4-D concentration group; HCG: high 2,4-D concentration group. (HCG, MCG) x (SG, LCG): *p* < 0.05

**Fig. 3:**
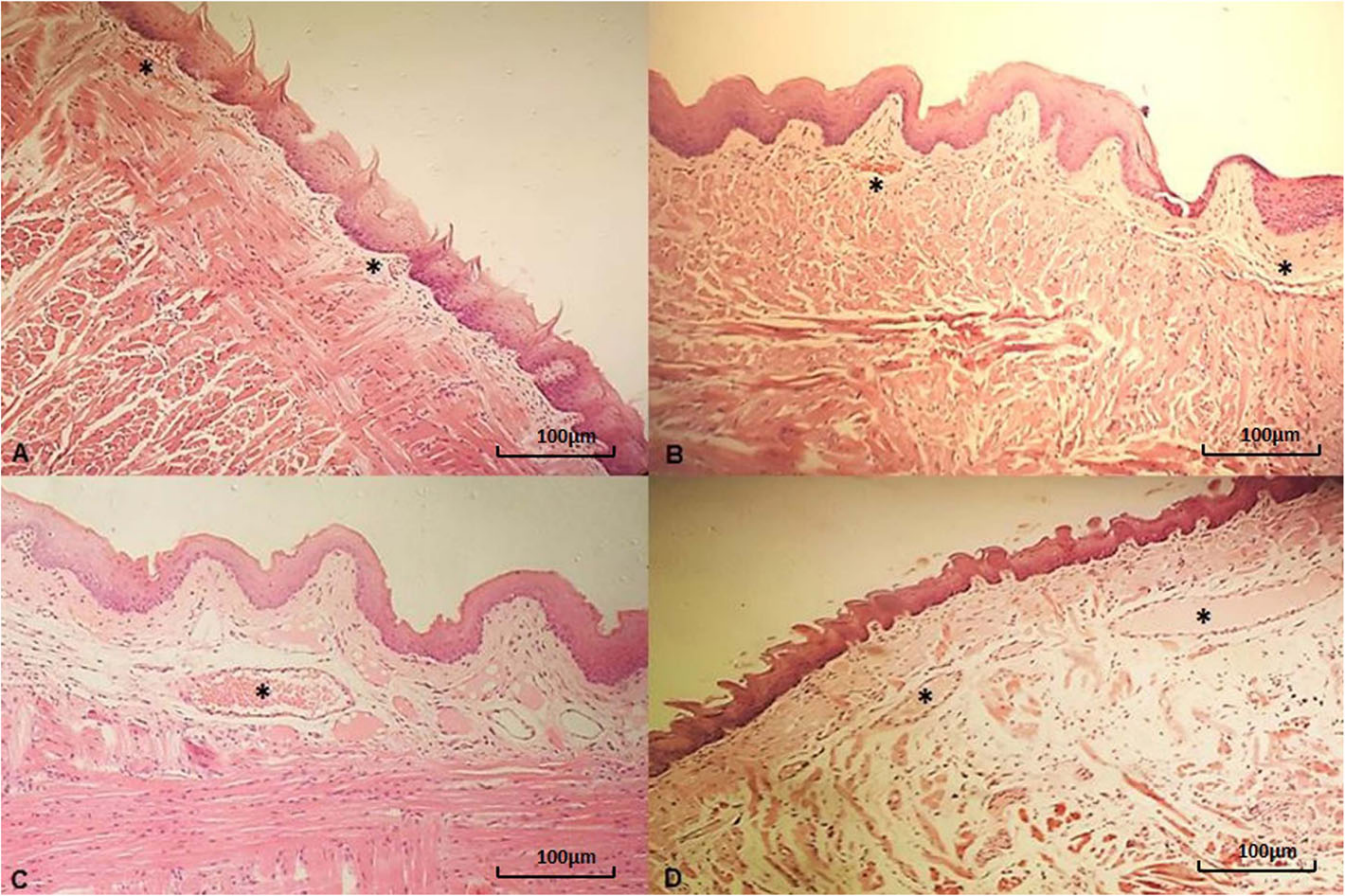
Photomicroscopy of the tongue with congestion. Note the dilated capillaries that are full of plasma and/ or red blood cells (*): **a** – Slightly dilated capillaries featuring mild tissue congestion (Animal of the SG group after 1 inhalation exposure of 2,4-D). **b** - Slightly dilated capillaries featuring mild tissue congestion (Animal of the LCG group after 1 inhalation exposure of 2,4-D). **c** - Very dilated capillaries featuring moderate tissue congestion (Animal of the MCG group after 3 inhalation exposures of 2,4-D). **d** - Very dilated capillaries featuring moderate tissue congestion (Animal of the HCG group after 2 inhalation exposures of 2,4-D). Hematoxylin-eosin, 100x magnification (100 µm bar for scale)

There was a statistically significant difference in the intensity of tissue congestion between the exposure concentrations (*p* = 0.002), but there was no difference when the exposure times were compared (*p* = 0.949) (Table 1; Figs. 2 and 3).

**Table 1:** Intensity of congestion, mean (± standard deviation) number of mast cells (per mm^2^), mean (± standard deviation) epithelial thickness of the dorsum and the ventral surface (in microns) and mean (± standard deviation) of the number of nucleoli organizing regions (NORs) in the epithelium of the dorsal and ventral surfaces of the tongue by study group and exposure time (*n* = 80)

Groups*	**Congestion**		**Number of mast cells**	**Epithelial thickness**		**Number of NORs**	
	**mild**	**moderate**		**dorsum**	**ventral surface**	**dorsum**	**ventral surface**
**SG 1D**	5/5 (100%)^a^	0/5 (0%)^a^	51.20 (15.04)^a^	34.55 (9.86)^a^	22.44 (4.23)^a^	396.6 (3.78)^a^	394.2 (13.55)^a^
**SG 2D**	5/5 (100%)^a^	0/5 (0%)^a^	53.00 (8.65)^a^	27.85 (7.87)^a^	27.58 (9.28)^a^	398 (12.57)^a^	374 (11.00)^a^
**SG 3D**	5/5 (100%)^a^	0/5 (0%)^a^	57.4 (11.13)^a^	30.86 (2.76)^a^	28.50 (17.07)^a^	405.8 (20.27)^a^	378.8 (5.40)^a^
**SG 8D**	5/5 (100%)^a^	0/5 (0%)^a^	62.2 (14.86)^a^	27.71 (6.18)^a^	21.73 (1.99)^a^	425.4 (20.18)^a^	359.8 (5.76)^a^
**LCG 1D**	5/5 (100%)^a^	0/5 (0%)^a^	57.4 (15.72)^a^	35.11 (5.03)^b^	24.61 (6.84)^a^	487.2 (6.97)^b^	377.8 (10.18)^a^
**LCG 2D**	5/5 (100%)^a^	0/5 (0%)^a^	57 (16.30)^a^	28.88 (4.75)^a^	24.02 (1.80)^a^	479.6 (11.90)^b^	363.4 (10.35)^a^
**LCG 3D**	5/5 (100%)^a^	0/5 (0%)^a^	63.6 (11.55)^a^	33.61 (6.11)^a^	25.56 (5.81)^a^	474.6 (13.93)^c^	384.8 (5.97)^a^
**LCG 8D**	5/5 (100%)^a^	0/5 (0%)^a^	46.2 (10.48)^a^	36.70 (4.60)^b^	29.70 (6.21)^a^	467.6 (6.18)^c^	394.2 (9.88)^a^
**MCG 1D**	5/5 (100%)^a^	0/5 (0%)^a^	39.6 (12.03)^b^	35.52 (4.16)^b^	25.32 (5.44)^a^	517 (21.62)^d^	513.4 (4.21)^b^
**MCG 2D**	5/5 (100%)^a^	0/5 (0%)^a^	59.8 (14.13)^a^	37.21 (7.29)^b^	28.68 (4.54)^a^	508.4 (13.46)^d^	506.2 (11.54)^b^
**MCG 3D**	2/5 (40%)^b^	3/5 (60%)^b^	49.6 (10.82)^a^	36.20 (3.10)^b^	20.03 (3.03)^a^	506 (5.33)^d^	494 (11.00)^b^
**MCG 8D**	5/5 (100%)^a^	0/5 (0%)^a^	50.8 (17.20)^a^	31.98 (8.73)^a^	29.24 (5.01)^a^	467.6 (1.34)^c^	394.2 (19.29)^a^
**HCG 1D**	3/5 (60%)^b^	2/5 (40%)^b^	42.6 (15.63)^b^	24.07 (5.64)^c^	23.44 (8.22)^a^	405.6 (28.93)^a^	403.2 (22.86)^c^
**HCG 2D**	3/5 (60%)^b^	2/5 (40%)^b^	40.2 (5.27)^b^	30.81 (8.47)^a^	27.70 (6.42)^a^	461 (13.13)^c^	387.2 (21.56)^a^
**HCG 3D**	5/5 (100%)^a^	0/5 (0%)^a^	44 (12.18)^b^	38.41 (4.52)^b^	26.76 (3.52)^a^	444.8 (20.46)^d^	410 (27.16)^c^
**HCG 8D**	3/5 (60%)^b^	2/5 (40%)^b^	32.2 (12.98)^b^	35.97 (3.22)^b^	23.93 (5.22)^a^	466 (10.93)^c^	377.2 (11.71)^a^

*Groups: *SG* saline group; *LCG* low 2,4-D concentration group; *MCG* middle 2,4-D concentration group; *HCG* high 2,4-D concentration group; *1D* one nebulization; *2D* two nebulizations; *3D* three nebulizations; *8D* eight days after three nebulizations. Different lowercase letters means *p*-value < 0.05, where: a ≠ b, c, d; b ≠ a, c, d; c ≠ a, b, d; and d ≠ a, b, c. Lowercase letters compare the groups at the same time and in the same column

**Fig. 4:**
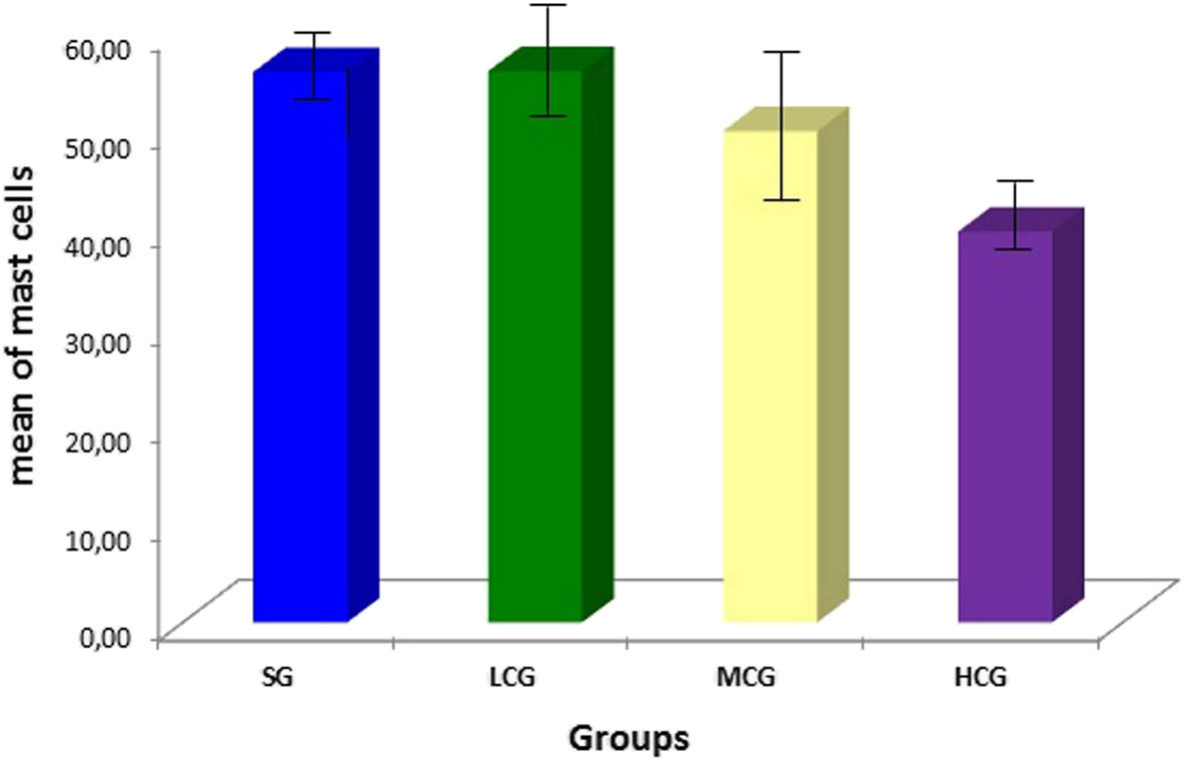
Mean number (± standard error) of mast cells (per mm^2^) per study group regardless the time of exposure. Groups: SG saline group, LCG low 2,4-D concentration group, MCG middle 2,4-D concentration group, HCG high 2,4-D concentration group. HCG x (SG, LCG, MCG): *p* < 0.05

**Fig. 5:**
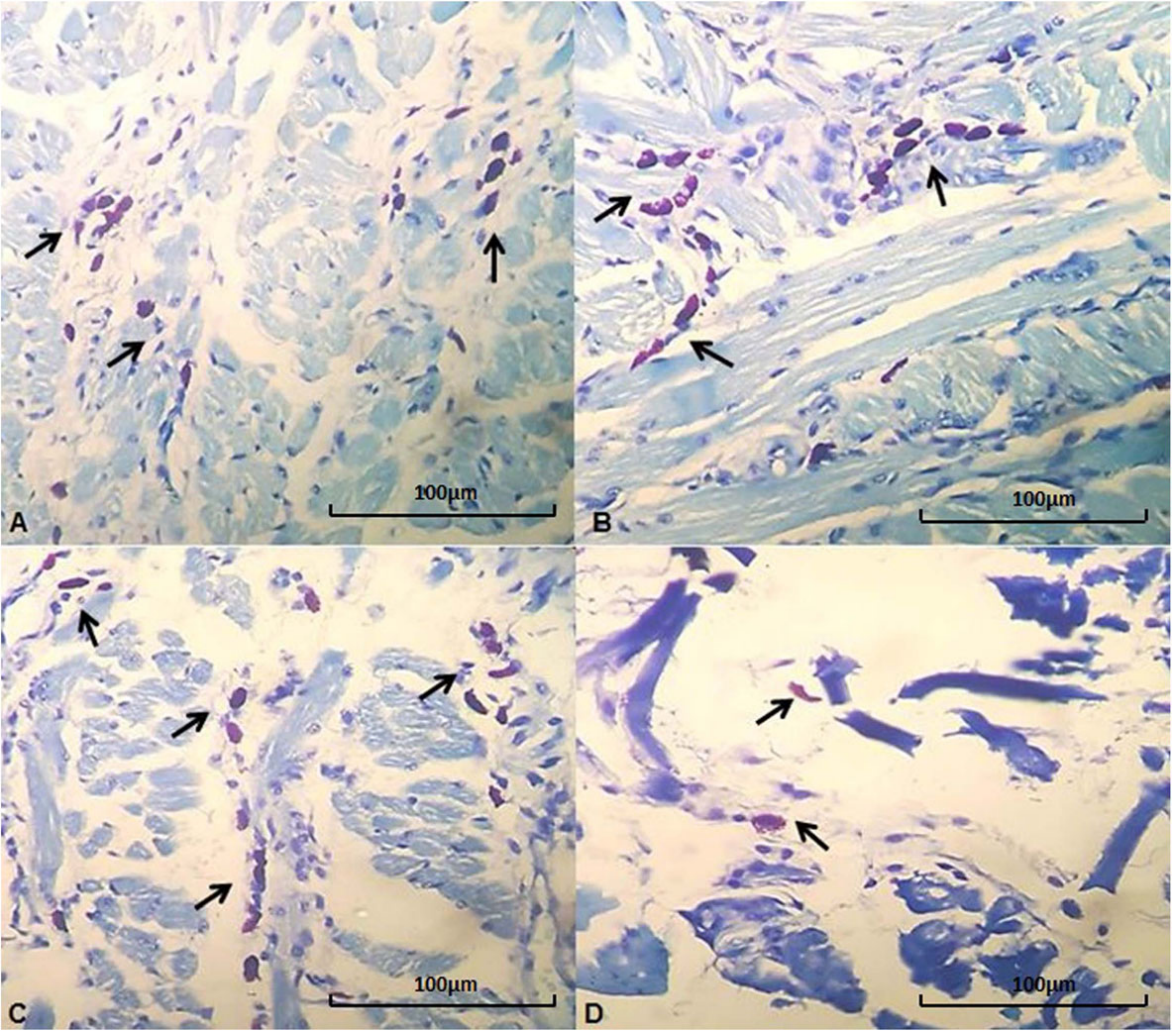
Photomicroscopy of the tongue showing mast cells (arrows). **a** - Large number of mast cells in the tissue (Animal of the SG group after 2 inhalation exposures of 2,4-D). **b** - Large number of mast cells in the tissue (Animal of the LCG group after 2 inhalation exposures of 2,4-D). **c** - Large number of mast cells in the tissue (Animal of the MCG group after 2 inhalation exposures of 2,4-D). **d** - Few mast cells in the tissue (Animal of the HCG group eight days after three nebulizations of 2,4-D). Hematoxylin-eosin, 400x magnification (100 µm bar for scale)

There was a statistically significant difference in mast cell counts between the exposure concentrations (*p* = 0.002), but there was no difference in relation to exposure times, nor was there an interaction between the exposure concentrations and exposure times (*p* = 0.450). The HCG group differed from the other groups (*p* < 0.05), but no difference in exposure times was observed (*p* = 0.418) (Table 1; Figs. 4 and 5).

### Measurement of epithelial thickness

The mean measurements of the dorsal epithelium thickness differed from those of the ventral surface, with a greater thickness in the dorsum (*p* < 0.001). There was no correlation between the mean thickness of the dorsal epithelium and the mean epithelial thickness of the ventral surface (*r* = 0.42; *p* = 0.711).

On the dorsum of the tongue, there was a statistically significant difference in the interaction between the exposure concentrations and exposure times (*p* = 0.013) but not in relation to exposure concentrations (*p* = 0.351) or exposure times (*p* = 0.423) alone (Table 1).

There was no difference in the thickness of the ventral surface epithelium between the exposure concentrations (*p* = 0.976), exposure times (*p* = 0.543), or the interaction between exposure concentrations and exposure times (*p* = 0.308) (Table 1).

### Number of Nucleoli Organizing Regions (NORs)

The mean number of NORs in the dorsum was different from that of the ventral surface, and it was higher in the dorsum (*p* < 0.001). There was a significant correlation between the mean NOR count in the dorsum and the mean NOR count in the ventral surface (*r* = 0.605, *p* < 0.001).

On the dorsum of the tongue, there were statistically significant differences between the exposure concentrations (*p* < 0.001), exposure times (*p* < 0.001) and the interaction between exposure concentrations and exposure times (*p* = 0.017). This pattern was also observed for the ventral surface, where there was a statistically significant difference between the exposure concentrations (*p* < 0.001), exposure times (*p* = 0.001) and the interaction between exposure concentrations and exposure times (*p* = 0.001). However, on the ventral surface, there was no difference between the SG and LCG groups (*p* = 0.458) (Table 1).

### Correlation between epithelial thickness and number of Nucleoli Organizing Regions (NORs)

**Fig. 6:**
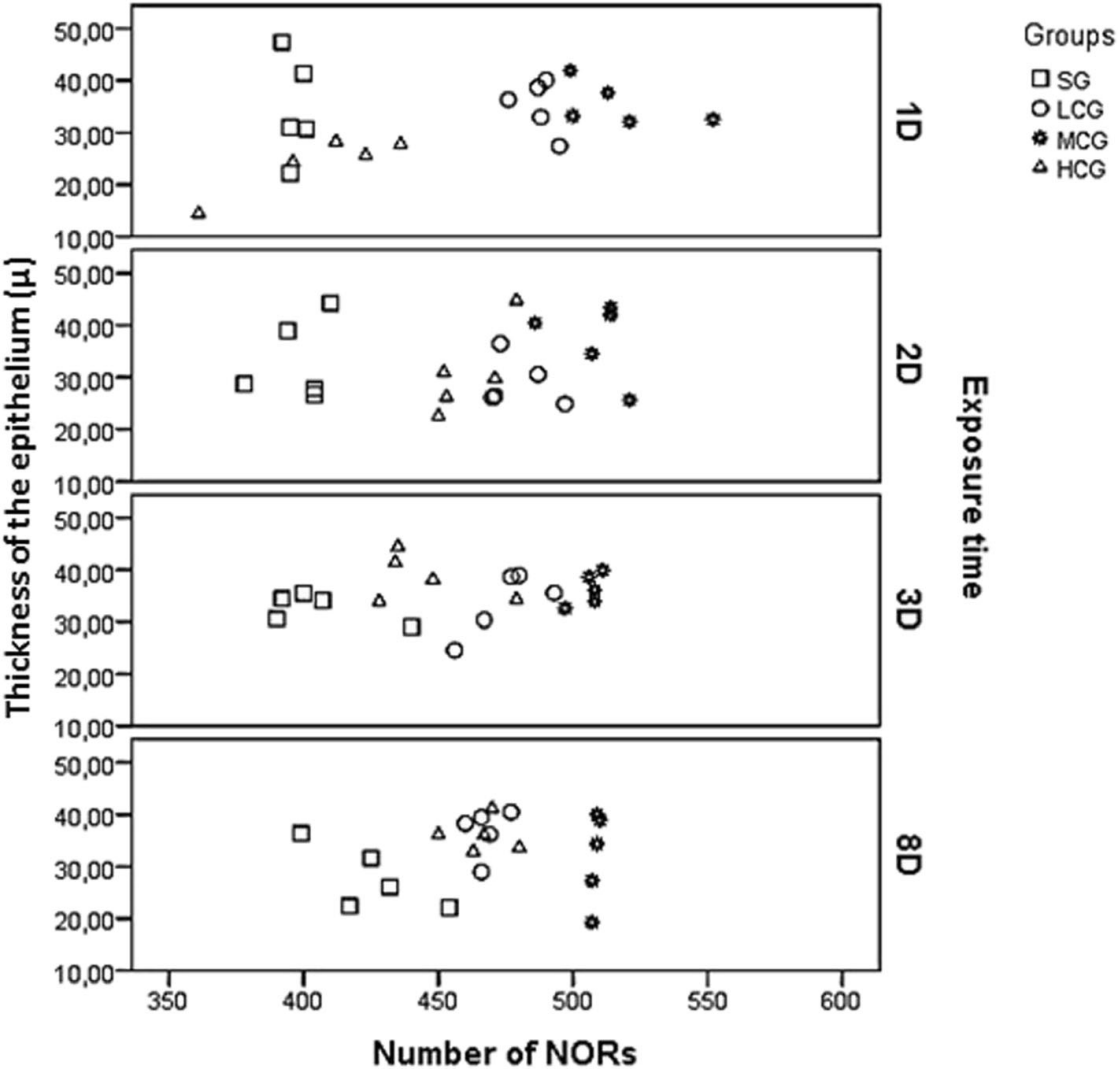
Correlation between the epithelial thickness (in microns) and the number of nucleoli organizing regions (NORs) (per mm^2^) in the epithelium of the dorsum of the tongue by study group and time of exposue (*p* = 0.048). Groups: SG: saline group; LCG: low 2,4-D concentration group; MCG: middle 2,4-D concentration group; HCG: high 2,4-D concentration group; 1D: one nebulization; 2D: two nebulizations; 3D: three nebulizations; 8D: eight days after three nebulizations

There was a significant correlation between the epithelial thickness and the number of NORs in the dorsum of the tongue (*r* = 0.222; *p* = 0.048) (Fig. 6) but not in the ventral surface (*r* = 0.008; *p* = 0.947).

## Discussion

In this study, acute exposure to high doses of 2,4-D caused an increase in proliferation index and in the thickness of the epithelium of the dorsum of the tongue and stimulated early tissue inflammatory response (congestion). But, there was a decrease in the number of mast cells in the tongue of the group exposed to high concentration of 2,4-D.

Occupational exposure to 2,4-D may occur during the production of this herbicide or through its agricultural, forestry or lawn use. Occupational exposure to 2,4-D characteristically occurs through inhalation or dermal absorption. Indirect or paraoccupational exposure may occur in some populations as a result of drift. Exposures to the general population may result from 2,4-D that is present in household dust, food, air, water, or soil. In some areas, residential exposures may be related to the use of 2,4-D in lawns [6].

Drift occurs when agricultural spraying is carried away from the target area by the wind and is thus an important source of environmental contamination [7]. Due to this contamination, the United States Environmental Protection Agency (EPA) has established restrictions to prevent the drift of pesticides to include an area of 9,144 m around the application area that is not sprayed, no pesticide application when the wind speed is greater than 15 miles per hour, and permission only for terrestrial applications [8]. Despite these recommendations, poor application conditions favor the formation of drift, leading to contamination not only for those handling the pesticide but also in the resident population of areas that are close to and far from agricultural spraying areas. Thus, inhalation exposure becomes an important route of contamination both occupationally and paraoccupationally and may lead to damages not only to the respiratory tract but also to the oral cavity.

A study of workers who produce chlorophenoxide herbicides observed increased keratinization of the epithelium of the buccal mucosa of these workers [5]. In the present study, hyperkeratosis was not observed, likely because this study included an acute exposure and hyperkeratosis implies an adaptive alteration of the squamous epithelium that occurs after a constant and long-lasting insult. However, we observed an increased thickness of the dorsal epithelium of the tongue, and this increase was also observed as an interaction between the concentration of the herbicide and the exposure time. No change in the epithelium of the ventral surface was observed between the study groups. These data show that the epithelium of the dorsum is most affected by the herbicide 2,4-D, and this increase in thickness may be a reactive response to the cellular damage that is caused by this herbicide. The epithelium of the ventral surface of the tongue is likely more protected from the effects of 2,4-D due to the cleansing substances in this area from the release of saliva by the sublingual salivary glands.

The NORs (nucleolar organizing regions) are regions that consist of chromosome fragments, around which the nucleoli form at the end of mitosis. The evaluation of NORs has been investigated as a marker of cellular proliferation [9]. In this study, we observed an increase in the number of NORs that was greater in the dorsum than in the ventral surface in the groups that were exposed to 2,4-D. In the dorsum, there was a difference between the concentration of herbicide and the exposure time. In addition, the epithelial thickness of the dorsum of the tongue correlated with the number of NORs in this area. The increase of NORs in the dorsum of the tongue and the correlation between the epithelial thickness corroborates the hypothesis of cellular damage caused by 2,4-D and the epithelial proliferation in response to it. The animals in the MCG group showed the highest number of NORs on both the dorsal and the ventral surfaces of the tongue. We believe that this phenomenon may have occurred because the low concentration was not sufficient to cause substantial epithelial damage, and therefore there was no increase in NORs. The high concentration probably caused more severe damage to the epithelia, not allowing enough time for the increase in nuclear activity.

Previous studies have shown an increased occurrence of chromatin damage in the oral cavity cells of the inhabitants of the Bin Mi village (Shongbe Province, Vietnam), which was heavily bombarded with “Agent Orange” in the 1960s [4]. In addition, a higher incidence of nuclear abnormalities and the appearance of micronuclei has been observed in the buccal mucosa of workers who produce chlorophenoxide herbicides [5]. These alterations favor the appearance of dysplastic or neoplastic lesions in the buccal epithelium. In the present study, no dysplastic or neoplastic changes were observed in the tongue epithelium, likely due to the short 2,4-D exposure time (maximum of three exposures). Studies evaluating chronic exposure to this herbicide may better show the likelihood of developing dysplastic or neoplastic changes in the oral mucosa through exposure to 2,4-D.

A study of the organochlorine methoxychlor in BALB/c mice showed an increase in mast cell degranulation and, consequently, an increase in allergic symptoms [10]. In another study in BALB/c mice, 2,4-D-specific IgE antibodies were detected following an intraperitoneal administration of 2,4-D [11]. The study by Fukuyama, et al. [12] in BALB/c mice that were initially sensitized and later exposed by intratracheal administration of 2,4-D suggests that this herbicide is an allergen to the respiratory tract and can cause an inflammatory response and increase in the levels of immunoglobulin (Ig) E and induce an influx of eosinophils and neutrophils to this area. A subsequent study showed that 2,4-D may be a more potent allergen and an inducer of inflammation if there is a simultaneous exposure to other pesticides [13]. In our study, there was no difference in the intensity of inflammation between the 2,4-D-exposed and unexposed groups. These data from short-term exposures may explain why we not have observed a significant inflammatory process in the groups that were exposed to 2,4-D. However, in contrast to other studies of the respiratory tract, there was a decrease in the number of tongue mast cells in the animals that were exposed to high doses of 2,4-D, showing the potential for inhibition of the inflammatory cell response of this herbicide or a direct immunotoxic effect of 2,4-D to inflammatory cells when used in high doses. There is moderate evidence to suggest that 2,4-D causes immunosuppression [6]. These effects of 2,4-D exposure on the proliferation of lymphocytes in humans are contradictory, since suppressive and stimulatory effects have been demonstrated, depending on the exposure levels and the formulation of 2,4-D [6]. Rats and mice also show conflicting results, with some studies showing immunosuppression [14, 15] and others showing an increased colony formation capacity, activation of hematopoiesis and migration of monocytes into the peripheral blood [16].

Vasodilatation and increased blood flow to tissues (tissue congestion) are the first changes that can be observed in acute inflammation [17]. In this study, we observed more marked tissue congestion in the animals of the MCG and HCG groups. These data show that at higher concentrations, regardless of the number of exposures, 2,4-D stimulates the initial inflammatory reaction in the tissues, although we did not find any significant leukocyte infiltrate in the tissues.

The effects of the herbicide 2,4-D have been poorly evaluated after short-term or acute exposures and have instead focused more on chronic exposure, since this is the more common method of exposure. However, even with a short duration (of three exposures or less), acute and high concentrations of 2,4-D inhalation exposures resulted in damage to the buccal epithelium, as well as the respiratory epithelium, which has been demonstrated in other studies.

Studies evaluating chronic exposure to 2,4-D can show whether there is a decrease in the inflammatory cell response or even in the appearance of dysplastic and / or neoplastic cells in the buccal mucosa. Furthermore, the evaluation of other areas of the buccal mucosa in addition to the tongue and the use of other diagnostic methods, such as exfoliative cytology for sample enlargement, may show changes other than those that can be observed in the tongue. In addition, the evaluation of oral exposure to the herbicide 2,4-D, will also be important for the proper analysis of the effects of this herbicide to the buccal mucosa.

## Conclusions

With the data from this study, we conclude that even after an acute exposure to 2,4-D there is an increase in the proliferation index and in the thickness of the epithelium in the dorsum of the tongue that likely occurs as a response to the cellular damage caused by this herbicide. Although 2,4-D stimulates an early inflammatory response (congestion) in tissues at higher concentrations, a decrease in the number of mast cells was also observed in the tongues of the groups that were exposed to high concentrations of 2,4-D, possibly indicating an inhibition of inflammatory cell response by high concentrations of this herbicide. The observed changes were associated with the dose of 2,4-D herbicide and not with the number of nebulizations.

## Methods

### Animal protocol

The methodology that was used in this study was first described by Mello, et al. [18].

The animals included in the study were male Swiss mice weighing between 30 and 45 g and in good health. Animals that did not meet these criteria were excluded from the study before it began.

We used 80 Swiss male mice (30–45 g), provided by the Central Vivarium of the Universidade do Oeste Paulista (UNOESTE), that were allocated in cages measuring 30 × 16 × 19 cm (5 animals per cage) and maintained in a room with a controlled temperature of 25 ± 2 °C, a relative humidity of 50 ± 15%, and a normal photoperiod (12–12 h light-dark cycle).

To determine the minimum sample size for comparisons between the groups, the “pwr” package in R was used to calculate sample sizes for conducting analysis of variance [19], where the test power = 80%, significance level = 5%, number of groups to be compared = 16 (4 exposure groups x 4 exposure times), and effect size (Cohen’s d) = 0.50. From these data, it was concluded that at least 5 elements per group were necessary.

The animals were randomly divided by drawing of lots into the following four groups (*n* = 20):

- SG, saline group: exposed to nebulization of 10 ml of 0.9% sodium chloride solution.
- LCG (low 2,4-D concentration group): exposed to herbicide mist with 3.71 × 10^− 3^ g of active ingredient per hectare (g.i.a. / ha) (4.6 µl of the pesticide was added to saline), corresponding to 187,17 mg/m^3^ of 2,4-D.
- MCG (middle 2,4-D concentration group): exposed to nebulization of the herbicide with 6.19 × 10^− 3^ g.i.a./ha (7.7 µl of pesticide was added to saline), corresponding to 313,31 mg/m^3^ of 2,4-D.
- HCG (high 2,4-D concentration group): exposed to nebulization of the herbicide with 9.28 × 10^− 3^ g.i.a./ha (11.5 µl of pesticide was added to saline), corresponding to 467,93 mg/m^3^ of 2,4-D.

The different concentrations of the herbicide 2,4-D were diluted in 10 ml of 0.9% sodium chloride to perform the nebulization. The solutions were prepared at the time of use.

- The different concentrations of the 2,4-D herbicide were formulated based on the product label, which shows the different herbicide concentrations for each type of crop to be sprayed, and a dose-adjustment was made to the box area to simulate environmental occupational exposure.

### 2,4-D herbicide exposure protocol

The mice were exposed to the herbicide dichlorophenoxyacetic acid (Nortox SA, Arapongas, Paraná, Brazil), which had the following composition: (2,4-dichlorophenoxy) acetic acid (2,4-D) dimethylamine salt: 806 g / liter (80.6% w / v), acid equivalent of 2,4-D: 670 g / liter (67.0% w / v) and inert ingredients: 424 g / liter (42.4% w / v). Handling of the 2,4-D herbicide was performed with the following personal protective equipment: rubber gloves, goggles and filter masks for gases.

The nebulization protocol consisted of two boxes (32 × 24 × 32 cm), and each box was attached to a Pulmosonic Star® ultrasonic nebulizer (Soniclear Ind. Com. Imp. and Exp. Ltda., São Paulo, Brazil) [18]. The exposure time was approximately 15 min.

Five animals from each group were exposed to nebulization at different times and were thus identified:

- 1D: one nebulization;
- 2D: two nebulizations on consecutive days with a 24 h difference between each exposure;
- 3D: three nebulizations on consecutive days with a 24 h difference between each exposure.

**Fig. 7:**
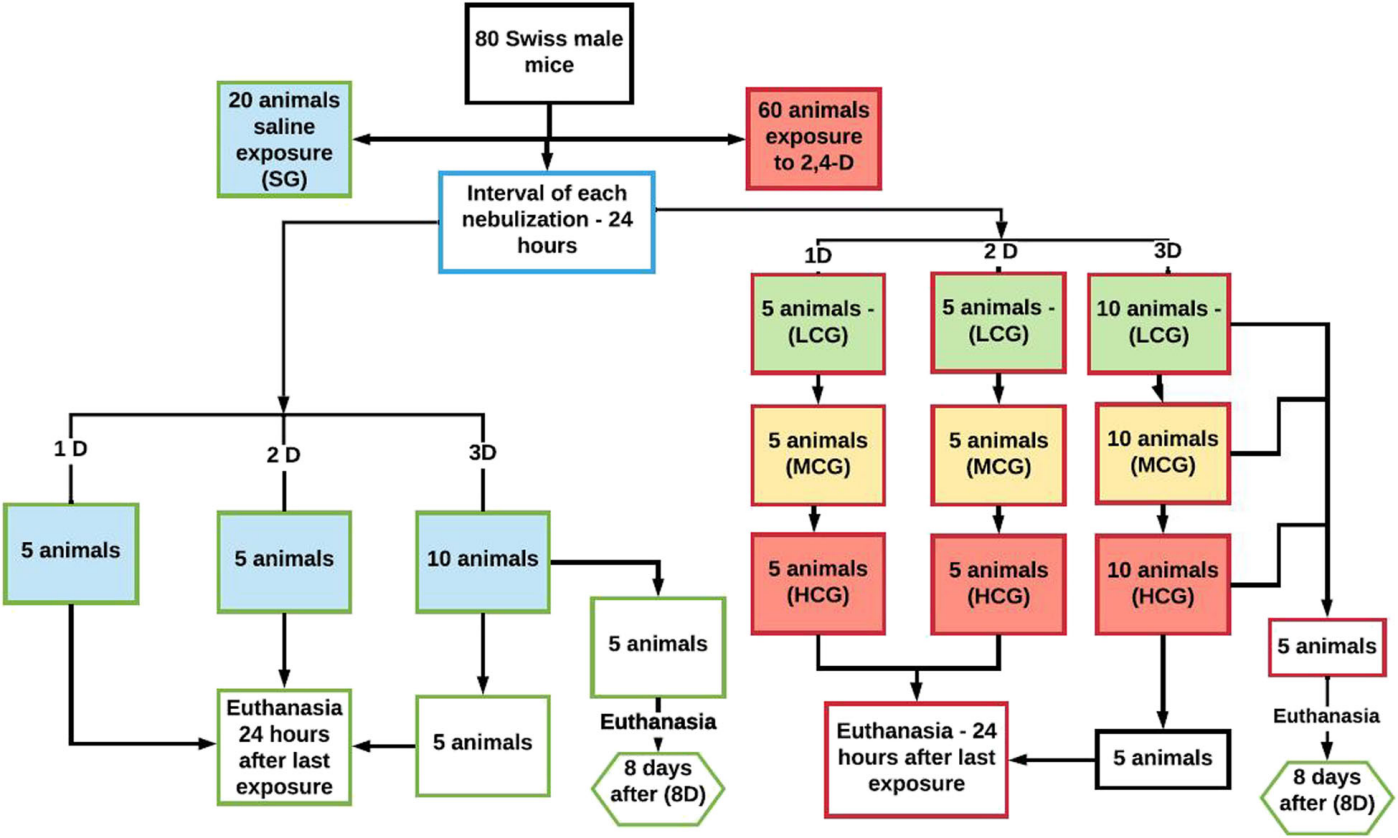
Experimental study design. SG: saline group; LCG: low 2,4-D concentration group; MCG: middle 2,4-D concentration group; HCG: high 2,4-D concentration group; 1D: one nebulization; 2D: two nebulizations; 3D: three nebulizations; 8D: eight days after three nebulizations

The animals were euthanized 24 h after the last nebulization, and five of the animals from the group that was nebulized three times were euthanized eight days (8D) after the last nebulization (Fig. 7).

Euthanasia was performed with an intraperitoneal injection of sodium thiopental at a dose of 100 mg / kg body weight [20]. After euthanasia, the entire tongue was removed for the histopathological analysis.

### Histopathological analysis

The tongue was longitudinally sectioned and fixed in 10% buffered formalin (Kinetics Chemical Industry, São Paulo, Brazil) for 24 h, and the samples were submitted for normal histological processing by embedding the tissues in paraffin (Dynamic Analytical Reagents, São Paulo, Brazil). Serial sections of 5 µm were obtained and stained by the hematoxylin-eosin (HE) method (Dolles, São Paulo, Brazil).

The investigator who conducted the histopathological analysis was blinded and evaluated the following types of tongue lesions: congestion (0 = absent, 1 = mild, 2 = moderate, 3 = marked); presence of hyperkeratosis (0 = absent, 1 = mild, 2 = moderate, 3 = marked); presence of parakeratosis (0 = absent, 1 = focal, 2 = diffuse); presence and intensity of polymorphonuclear and / or mononuclear inflammatory infiltrate (0 = absent, 1 = discrete, 2 = moderate, 3 = intense); presence of individual cell necrosis (0 = absent, 1 = present); preneoplastic or dysplastic lesions (0 = absent, 1 = mild, 2 = moderate, 3 = severe dysplasia); and benign neoplastic lesions or malignant neoplastic lesions [21].

The tongue sections were also stained by Toluidine blue (Merck, Darmstadt, Germany) for the identification of mast cells. The number of mast cells was counted in the lamina propria in 10 high power fields (HPF), corresponding to approximately 1 mm^2^ [22].

The measurements of tongue mucosa thickness were performed in two areas each [21] of the dorsal and ventral surfaces of the tongue in all animals at 200x magnification using ImageJ® software from the National Institutes of Health (NIH), available free of charge on the Internet (http: //rsbweb.nih.gov/ij/).

To count the NORs (nucleoli organizing regions), other deparaffinized sections of the tongue were stained by silver impregnation according to Ploton, et al. [23]. The counting pattern of the NORs was performed considering 10 cells / HPF, repeating in 10 random microscopic fields to total the count in 100 cells per animal [23]. The NORs were separately counted in the dorsal and ventral surfaces of the tongue.

### Statistical analysis

For the qualitative variables, the likelihood ratio was calculated, and a two-way analysis of variance was used to analyze the quantitative variables. The correlation between the quantitative variables was also used. The level of significance was set at 5%, and SPSS V.22 software was used to perform the analyses.

## Data Availability

All data generated or analyzed during this study are included in this published article.

## Abbreviations

2,4,5-T: 2,4,5-trichlorophenoxyacetic acid
2,4-D: Dichlorophenoxyacetic acid
EPA: United States Environmental Protection Agency
HCG: High 2,4-D concentration group
HE: Hematoxylin-eosin method
HPF: High power field
Ig: Immunoglobulin
LCG: Low 2,4-D concentration group
MCG: Middle 2,4-D concentration group
NORs: Nucleoli organizing regions
SG: Saline group
